# Finite Element Analysis and Near-Infrared Hyperspectral Reflectance Imaging for the Determination of Blueberry Bruise Grading

**DOI:** 10.3390/foods11131899

**Published:** 2022-06-27

**Authors:** Zhaoqi Zheng, Zimin An, Xinyu Liu, Jinghui Chen, Yonghong Wang

**Affiliations:** 1 Tianjin Key Laboratory of Integrated Design and On-Line Monitoring for Light Industry & Food Machinery and Equipment, College of Mechanical Engineering, Tianjin University of Science & Technology, Tianjin 300222, China; anzimin8064@163.com (Z.A.); liuxinyu987321@163.com (X.L.); cjh05282022@163.com (J.C.); wang_yong_hong11@163.com (Y.W.); 2 Tianjin International Joint Research and Development Center of Low-Carbon Green Process Equipment, Tianjin 300222, China

**Keywords:** blueberry bruise damage, finite element, response surface, hyperspectral reflectance imaging, uniaxial compression experiment

## Abstract

Bruising of the subcutaneous tissues of blueberries is an important form of mechanical damage. Different levels of bruising have a significant effect on the post-harvest marketing of blueberries. To distinguish different grades of blueberry bruises and explore the effects of different factors, explicit dynamic simulation and near-infrared hyperspectral reflectance imaging were employed without harming the blueberries in this study. Based on the results of the compression experiment, an explicit dynamic simulation of blueberries was performed to measure the potential locations of bruises and preliminarily divide the bruise stages. A near-infrared hyperspectral reflectance imaging system was used to detect the actual blueberry bruises. According to the blueberry photos taken by the near-infrared hyperspectral reflectance imaging system, the actual bruise rates of blueberries were obtained by using the Environment for Visualizing Images software for training and classification. Bruise grades of blueberries were divided accordingly. Response surface methodology was used to determine the effects of ripeness, loading speed and loading location on the blueberry bruising rate. Under the optimized parameters, the actual damage rate of blueberries was 1.1%. The results provide an important theoretical basis for the accurate and rapid identification and classification of blueberry bruise damage.

## 1. Introduction

Blueberries are bilberry plants of the rhododendron family, native to North America and East Asia [1]. Blueberries are rich in more than 15 kinds of anthocyanins and a variety of vitamins, which have decent health care effects and high nutritional value [2,3]. Scientific literature shows that blueberries play an important role in maintaining blood sugar balance, preventing cardiovascular disease, anti-tumor activity, and more [4]. Consequently, blueberries have become popular worldwide and are recommended by the World Food and Agriculture Organization as one of the five healthiest fruits.

The quality of blueberries has a great impact on their economic value; however, blueberries will inevitably be mechanically damaged during the picking and transportation process [5]. Bruising is the main manifestation of blueberry mechanical damage [6,7,8,9]. Bruising occurs when the external force applied to the fruit exceeds the carrying capacity of the fruit cells. Usually, the skin of the fruit does not break at this time [10,11,12]. Bruised blueberries are extremely perishable and dehydrated [13] which can cause significant economic losses to the blueberry industry. Visual and destructive methods are commonly used to estimate the bruise grade of fruits. Vertical cuts along the long axis of the apple are used to measure major and minor width and depth, and the bruise area and volume are quantified to assess apple damage. [14]. Bruising caused by the vibration of bananas can be quantified by the transparent damage scale [15]. Damage reference charts are used to assist raters in grading the damage based on the standard damage score provided [16]. However, these methods are too subjective and relied primarily on manual direct observation of the damage to be assessed.

More rational research methods for fruit damage assessment have been developed. Fruit damage prediction is usually performed by the finite element method [17,18,19,20,21,22]. Experimental studies on cantaloupe damage reveal that internal contusion damage occurs when the total deformation is approximately 20%. The finite element method is used for prediction, and the correlation between the predicted and measured values is high, which proves that the finite element method can be used to predict the internal bruise damage of cantaloupe [23]. Drop mechanical properties of pears with different maturities can be determined by a combination of experiments and finite element simulation, and the bruised area of pears can be effectively predicted [24]. A multi-scale finite element model was used to analyze the damage to various structures inside the tomato under external force. Simulated data confirm the experimental results that mechanical damage first occurs in the compartmental gel tissue, effectively predicting mechanical damage inside the tomato [25]. On the other hand, visually displaying the damaged area is very important for the study of fruit mechanical damage, especially for damage assessment. The advanced non-destructive testing technology overcomes the disadvantage that the damage cannot be assessed with the naked eye or touch when the damage occurs inside the fruit, and also avoids the destructive testing of the fruit. Near-infrared hyperspectral imaging technology has become a tool for accurately measuring fruit quality, and has been applied to citrus [26], date [27], apple [28], olive fruit [29], litchi [30], and other fruits [31,32,33,34,35,36]. Early internal bruises of blueberries are detected by hyperspectral transmission imaging technology, and the bruised and healthy tissues are classified by a support vector machine. The stem tip location method is used to identify blueberries, and it is found that the average accuracy rate is as high as 94.5%. This test proves that near-infrared hyperspectral transmission imaging can be used to detect blueberry bruises within 30 min [37]. However, some studies found that reflectance imaging is better than transmission imaging to distinguish fresh blueberry lesions from average spectrum classification results for each fruit. The optimal wavelength and band ratio images among the three feature selection methods are used for bruise detection, and the overall identification of the CARS-LS-SVM model and the band ratio images are accurately displayed, which proves that the near-infrared hyperspectral reflectance imaging technique can detect the internal bruise of blueberries [38]. This provides an important detection technique for the assessment of blueberry bruises.

At present, the division of blueberry bruise damage is mostly by cutting the blueberries to observe the internal bruise area. When the damaged area is greater than 25%, it is considered that the blueberries have been damaged [39]. This method is destructive and subjective. Blueberry bruises were identified and assessed without harming the blueberries in this study, and the impacts of several factors on blueberry bruises were investigated. To initially predict the location as well as the extent of bruising in blueberries, ABAQUS was used to carry out the finite element explicit dynamic simulation. The near-infrared hyperspectral reflectance imaging technology was used to classify and count the damage to blueberries, and further grade the bruise degree of blueberries according to this. In addition, the response surface methodology was used to examine the effects of fruit maturity, load speed, and load location on the rate of fruit bruises. The research results could provide a theoretical basis for future research on the mechanical structure and mechanized processing process suitable for the picking, grading, packaging and transporting of blueberries.

## 2. Materials and Methods

### 2.1. Fruit Materials

“Blue Gold” blueberries taken from Shenyang, Liaoning Province, China, were used in this study. Based on the experience of fruit farmers, priority was given to picking quality fruit without obvious mechanical or insect damage. Individuals with poorer forms of fruit and great differences in body shape were eliminated. Fresh blueberries were transported to the laboratory at a low temperature and stored at 0 °C with 85% moisture. The bruise damage to blueberries from the transport process and the influence of environmental changes on the experiment were not considered. Before the experiment, the blueberries were taken out of the constant temperature and humidity box and placed at room temperature (23 °C, 50–55% RH) for 2 h so that the blueberries returned to room temperature.

### 2.2. Blueberry Physical Parameters

Fruit sphericity is one of the important physical characteristics and quality indicators of some commercial fruits [40]. A good form of fruit can better meet the needs of the market as well as the mechanical treatment and classification of packaging. The fruit sphericity of blueberry samples required for the study would be similar:

(1) $$G=\frac{L}{D}\;$$

where *G* is the fruit’s shape index, *L* is the longitudinal diameter (mm), and *D* is the horizontal diameter (mm).

Fruit density is also one of the important characteristics of fruit quality. Thirty blueberry samples were selected, and their quality was measured by electronic balance (CP224C, OHAUS, Shanghai, China). Blueberry volume was obtained by draining. Then, the density of blueberries was calculated by Equations (2) and (3):

(2) $$\Delta V=V_{1}-V_{0}\;$$

(3) $$\rho =\frac{M}{\Delta V}$$

where $V_{0}$ is the initial volume of water in the container (cm^3^), $V_{1}$ is the volume of water in the blueberry container (cm^3^), ΔV is the volume of the blueberries (cm^3^), *ρ* is the density (g/cm^3^), and *M* is the quality of the blueberries (g).

### 2.3. Blueberry Borderline Failure and Bruise Simulation

The Poisson’s ratio and elastic modulus required for blueberry finite element simulation can be achieved by compression experiment [41,42,43]. In the compression experiment of convex food materials, the moisture content has a great influence on the force-deformation characteristics, which are proposed in ASAE S368.4 DEC2000 (R2017). Hot-air drying is commonly used to determine the moisture content of the fruit [44,45,46,47]. The blueberry samples were cleaned and absorbed the surface moisture, and then placed on the material tray of the hot-air dryer box for drying. The hot-air speed was set to 1.2 m/s. The total weight of the sample and tray was weighed every 1 h, then promptly returned to the drying chamber after weighing. Each weighing time should not exceed 10 s until three consecutive weighing results were consistent. To prevent errors caused by decomposition and oxidation of certain components when heated above 100 °C, it was generally first baked at 60–70 °C to near dryness and then heated to 100–105 °C, to a constant weight. Afterward, the moisture content of the blueberries can be derived from Equation (4):

(4) $$X=\frac{m_{1}-m_{2}}{m_{1}-m_{3}}\times 100\%\;$$

where *X* is the moisture content of the sample (%), $m_{1}$ is the quality of the tray and the sample (g), $m_{2}$ is the mass of the tray and the sample after drying (g), and $m_{3}$ is the quality of the pallet.

The texture analyzer (CT3-4500, BROOKFIELD, Middleborough, MA, USA) was used to obtain data from compressed blueberries. A compression experiment was completed with the following settings: a trigger load of 7 g, a test speed of 0.1 mm/s, a pre-test speed of 2.00 mm/s, and a return speed of 3.00 mm/s. The diameter of the compression probe was 12 mm.

According to the longitudinal diameter of the blueberries, the compression distance of the probe was set reasonably. Blueberries were considered to be in a critical failure state when their skins begin to crack. At the moment of blueberry rupture, the load–distance curve decreased significantly. The data output of the texture analyzer recorded the compressed distance and load of the blueberries at this time. These data are borderline failure data for blueberries. The Poisson’s ratio and elastic modulus of blueberries need to be calculated by measuring the changing size and related parameters after the blueberries were compressed for a certain distance. In line with the provided method [48], Poisson’s ratio can be obtained by the following equations:

(5) $$\varepsilon_{x}=\frac{D_{0}-D_{1}}{D_{0}}$$

(6) $$\varepsilon_{Z}=\frac{L_{0}-L_{1}}{L_{0}}$$

(7) $$\nu =-\frac{\varepsilon_{x}}{\varepsilon_{z}}$$

where $D_{0}$ is the initial diameter (mm), $D_{1}$ is the diameter after the change (mm), $L_{0}$ is the initial longitudinal diameter (mm), $L_{1}$ is the longitudinal diameter after the change (mm), $\varepsilon_{x}$ is the transverse strain, $\varepsilon_{z}$ is the longitudinal strain, and ν is the Poisson’s ratio.

The elastic modulus can be obtained by the calculation formula mentioned in ASAE S368.4 DEC2000 (R2017), which is based on the Hertzian contact stress equation used in solid mechanics. A flat contact equation was used in the blueberry compression experiment:

(8) $$E=\frac{0.338F\left(1-\nu^{2}\right)}{D^{3/2}}{\left[K_{U}{\left(\frac{1}{R_{U}}+\frac{1}{R_{U}^{'}}\right)}^{1/3}+K_{L}{\left(\frac{1}{R_{L}}+\frac{1}{R_{L}^{'}}\right)}^{1/3}\right]}^{3/2}\;$$

where *E* is the elastic modulus (Pa), *D* is the deformation (m), ν is the Poisson’s ratio, *F* is the force (N), $R_{U}$ and $R_{L}$ are the minimum radius of curvature of the contact point between the sample and the upper and lower plates (m), $R_{U}^{\prime }$, and $R_{L}^{\prime }$ are the maximum radius of curvature of the contact point between the sample and the upper and lower plates (m). The constants $K_{U}$ and $K_{L}$ are determined by cosθ.

The value of cosθ can be obtained by the following equations:

(9) $$R_{1}=\frac{H}{2}\;$$

(10) $$R_{1}^{'}=\frac{H^{2}+L^{2}/4}{2H}\;$$

(11) $$cos\theta =\frac{\left[\frac{1}{R_{1}}-\frac{1}{R_{1}^{'}}\right]}{\left[\frac{1}{R_{1}}+\frac{1}{R_{1}^{'}}\right]}\;$$

where $R_{1}$ and $R_{1}^{\prime }$ are the minimum and maximum radius of the curvature radius of curvature, *H* is the height of the object, and *L* is the width of the object. The *K* can be obtained from the calculated θ by looking up the table below (Table 1).

**Figure 1:**
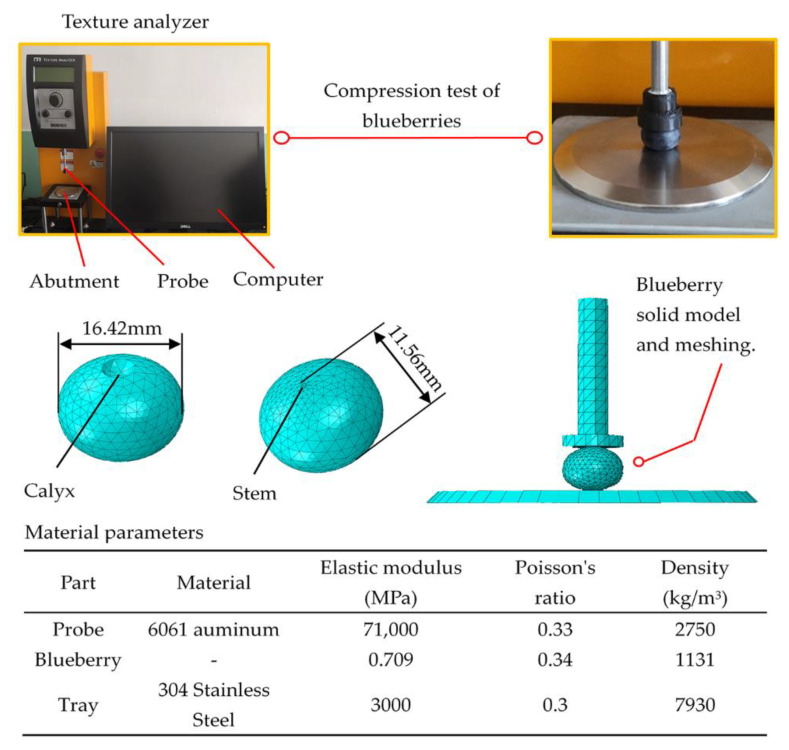
Blueberry compression and finite element model.

To simulate the blueberry bruises, suitable blueberries were selected to build a 3D model in SOLIDWORKS (Dassault Systemes, Paris, France) and imported into ABAQUS (SIMULIA, Providence, RI, USA) for finite element analysis. The assembly of the probe, tray, and blueberries took place in SOLIDWORKS. The required parameters of each component were entered in the property module. Step 1 in the module was set as dynamic and explicit. The nlgeom option was turned on, and the simulation time was set to 50 s. The contact types were defined in the interactive module, where normal contact was defined as hard contact, and tangential contact was defined as frictionless. The compression probe and tray were defined as rigid bodies. The compression speed of the probe was defined in the load module to be 0.1 mm/s and only moved on the *y*-axis, and the tray was completely fixed. In the mesh module, the approximate global size and maximum deviation factor of blueberries were set to 1.5 and 0.1, respectively. The approximate global dimensions of the tray and probe mesh were set to 5 and 3, respectively, with a maximum deviation factor of 0.1. In the simulation, 598 and 351 elements were generated from the compression probe and the tray, and 6529 elements were generated from the blueberries. The blueberry compression experiment and finite element model are shown in Figure 1.

### 2.4. Bruise Damage Detection Based on Hyperspectral Reflectance Imaging

**Figure 2:**
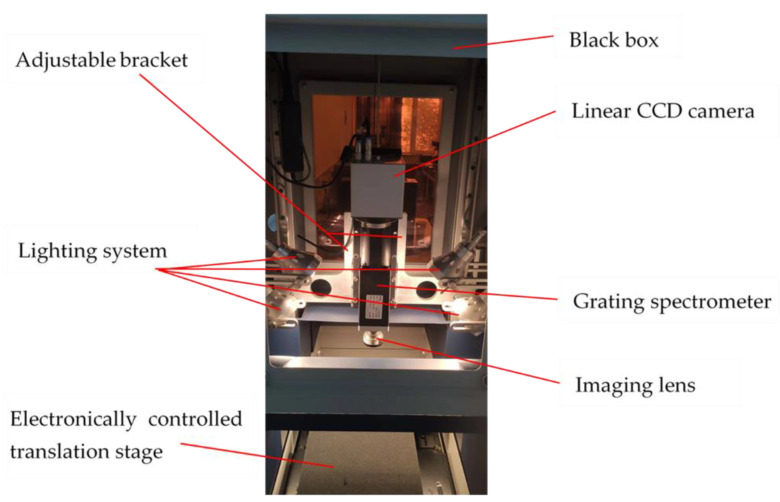
Near-infrared hyperspectral reflectance system.

A hyperspectral reflectance imaging sorter (Image-λ-N17E GaiaSorter, Zolix, Beijing, China) was used to collect blueberry images, as shown in Figure 2. This system consists of an illumination system mainly composed of a linear CCD camera (Zelos-258 GV, Kappa optronics GmbH, Reinhausen, Germany), a grating spectrometer (Imspector N17, Spectral Imaging, Oulu, Finland), a one-dimensional electronically controlled translation stage (PSA200-11-X, Zolix, Beijing, China), and four 35-W halogen lamps. The wavelength range that can be provided is 900–1700 nm. The system obtains a total of 256 bands in the wavelength range, so the spectral resolution should be 3 nm. A mercury lamp was used to calibrate the wavelength before the experiment began.

Before obtaining blueberry hyperspectral images, a white–dark reference should be used to calibrate the reflectance to eliminate the effects of changes in illumination intensity or the dark current of the camera. The white reference image was obtained by scanning polytetrafluoroethylene, and the black reference image was scanned by covering the camera lens. Finally, the original image was calculated using Equation (12) to obtain the corrected image.

(12) $$I=\frac{I_{0}-B}{W-B}\times 100\%\;$$

where $I_{0}$ is the originally acquired hyperspectral image, *B* is the black reference image, *W* is the white reference image, and *I* is the corrected hyperspectral image.

The correction process of the original image was carried out in SpectraVIEW (Isuzu Optics, China). After correction, a blueberry grayscale image was obtained and saved. Then, the image was imported into the Environment for Visualizing Images (ENVI) (Exelis Visual Information Solutions, Boulder, CO, USA) for further processing. According to previous scientific literature [49], the border between blueberries and the background was obvious at 1075 nm, and the blueberry damage area was darker than the healthy area at 1200 nm.

**Figure 3:**
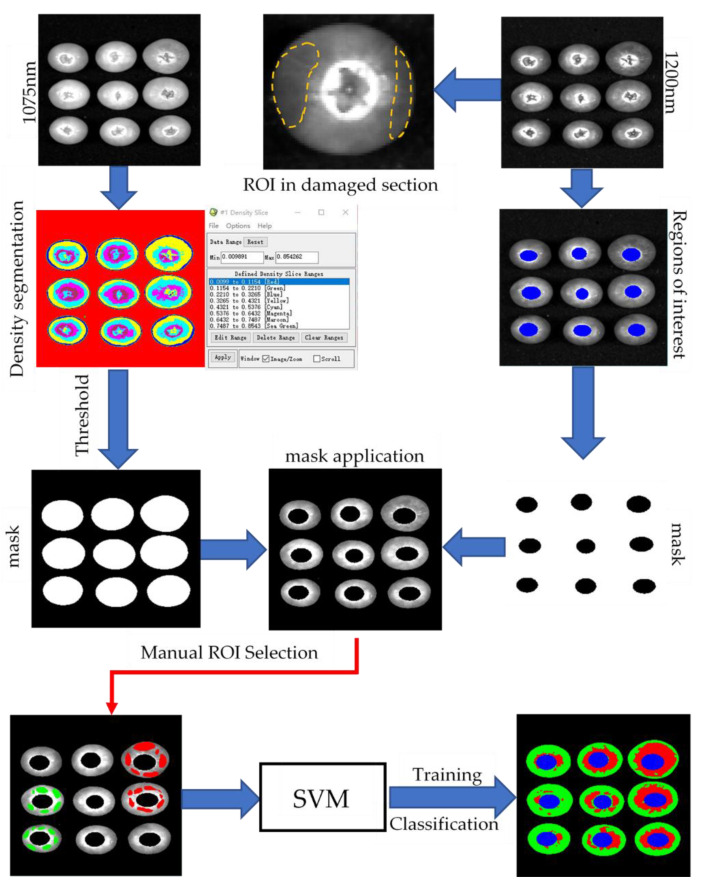
Hyperspectral image processing process of blueberries.

To separate blueberries from the background, the image was subjected to density segmentation at 1075 nm. At this time, the threshold between blueberries and the background was obtained. The obtained threshold was used in the production of the mask template, which was then applied to the original image. Fan et al. (2018) [50] proposed that the spectral characteristics of the calyx end were similar to the spectral characteristics of the damaged area, so to avoid the calyx area being identified as the damaged part, it was necessary to mask the calyx. The contour feature of the calyx was recognized at 1200 nm. The blueberry calyx outline was divided into regions of interest, and a mask was made accordingly. The hyperspectral image processing process of blueberries is shown in Figure 3.

In the 1200 nm band, the difference between bruised tissue and healthy tissue was obvious. Heathy and bruised regions were selected as regions of interest. The support vector machine (SVM) in ENVI was used for the training and classification of blueberry bruised and healthy tissues. The region of interest to be classified was selected. The radial basis function was called the kernel function, and the gamma value in the kernel function was the inverse of the selected band. The penalty parameter controlled the balance between the sampling error and the rigid extension of the classification. It was set to 100 and the pyramid levels were set to 0, so that the image was processed at the original resolution. The classification probability threshold was set to 0. If a pixel has all the rule probabilities calculated to be less than 0, then the pixel will not be classified. The above parameters were carried out under the guidance of experimental testing and SVM instructions under ENVI.

After the parameter setting was completed and the image saving path was set, the training and classification of the image would start. There would inevitably be some small spots in the image obtained after classification processing. Majority/minority analysis was used to remove small spots. This analysis used a method similar to convolution filtering to classify false pixels into a larger category within this category and then defined a transformation kernel size. The majority analysis replaced the center pixel category with the dominant pixel category in the transformation kernel. Minority analysis replaced the center pixel category with the category of the second pixel in the transformation kernel. The majority was chosen as the analysis method. The remaining parameters were set to default values. Finally, statistical tools were used to count the number of pixels in the blueberry damaged area. The bruise damage rate was calculated by the following equation:

(13) $$Y=\frac{S_{1}}{S_{1}+S_{2}}\;$$

where *Y* is the bruise damage rate (%), $S_{1}$ is the number of red pixels representing the damaged part, $S_{2}$ is the number of green pixels representing healthy parts.

### 2.5. The Influence of Some Factors on Bruise Damage

Fresh blueberries are extremely susceptible to mechanical damage during the picking, transportation, and sales process. Different influencing factors have different degrees of bruising damage to blueberries. In this experiment, blueberry maturity, loading location, and loading speed were explored. The optimal parameters of the three factors were determined to ensure the lowest damage rate for each blueberry. Blueberry maturity was divided into immature (1), nearly ripe (2), and mature (3). The whole immature blueberry fruit was reddish. Additionally, the stem end of the fruit that was close to maturity was red, while the calyx end and most of the equatorial area appeared dark. Ripe fruit tends to be darker. The blueberries were compressed at 0.1, 0.5 and 1 mm/s. Three loading locations were considered: the calyx (0°), the equator (90°), and the stem end (180°).

Design-Expert (Stat-East, Minneapolis, MN, USA) was used for response surface design. There were 17 sets of experiments. To reduce experimental error, each group of data was repeated 20 times, and an average value was obtained as the result. The bruising damage rates will affect the sales of blueberries, which have a significant impact on the economic value of blueberries. In this study, the hyperspectral reflectance system was used to detect the bruise degree of blueberries, and the bruise damage rates were used as the response variable to explore the influence of different influencing factors on the damage degree of blueberries.

## 3. Results and Discussion

### 3.1. Blueberry Parameters and Mechanical Simulation

Under the experimental conditions and calculation equations proposed in Section 2.2 and Section 2.3, the various parameters of blueberries were obtained. According to the blueberry geometric size measured by the vernier caliper, the fruit shape index of the blueberries was 0.704 by Equation (1). After the density determination test and the calculation of Equations (2) and (3), the density of the blueberries was 1131 kg/m^3^. A hot-air drying test was performed on blueberries, and the water content of blueberries was 89.94% by Equation (4). From Equations (5)–(7), the blueberry Poisson’s ratio was 0.34. The elastic modulus of the blueberries was 0.709 MPa according to Equations (8)–(11) and Table 1.

**Figure 4:**
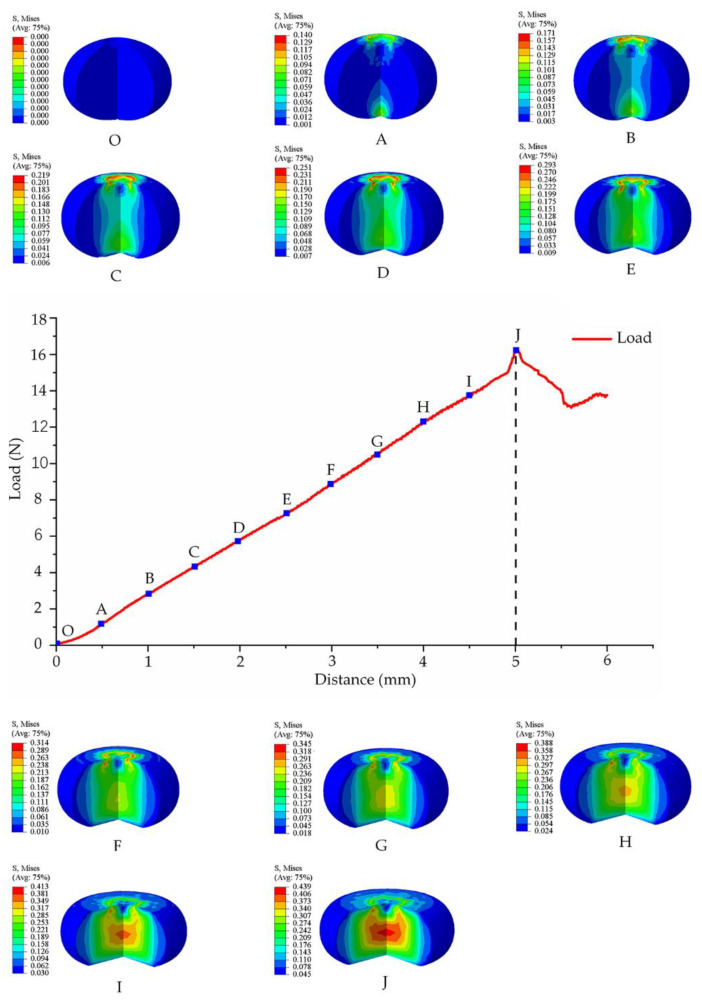
Blueberry load–distance curve and finite element simulation. Blueberry is compressed from O–J. The letters from O,A represent the stress state of blueberry at different compression distances, respectively. O: uncompressed blueberry; A: compression distance is 0.5 mm; B: compression distance is 1 mm; C: compression distance is 1.5 mm; D: compression distance is 2 mm; E: compression distance is 2.5 mm; F: compression distance is 3 mm; G: compression distance is 3.5 mm; H: compression distance is 4 mm; I: compression distance is 4.5 mm; J: compression distance is 5 mm.

Based on the blueberry compression experiment, the maximum compression of blueberry rupture was 5 mm and the maximum load was 16.22 N. The load acting on the blueberries changed when the fruit was deformed, as shown in Figure 4. As the distance increased, the load also increased. The blueberries started to burst when the curve reached point J. Then, ABAQUS was used to perform a mechanical simulation of the bruise damage to blueberries before the compression distance reached point J. The stress magnitude and distribution of blueberries were obtained. At point F, there was a significant stress mutation at the center of the blueberries. It was preliminarily inferred that blueberries were mildly damaged before point F and then severely damaged. The stress distribution of the blueberries was mainly concentrated on the calyx and stem end in the initial stage. These two locations were where the blueberries first came into contact with the probe and tray. As the compression distance increased, the blueberry stress distribution began to extend the center column. The stress at the equator was relatively small and did not change significantly. When the blueberries approached their maximum deformation, the stress at the equator changed significantly. According to the distribution and magnitude of the stress inside the blueberries, it was speculated that the blueberry bruise damage should first occur in the calyx and stem end, then extend to the central pillar, and finally, spread to the equator.

### 3.2. Bruise Damage Standard Division

**Figure 5:**
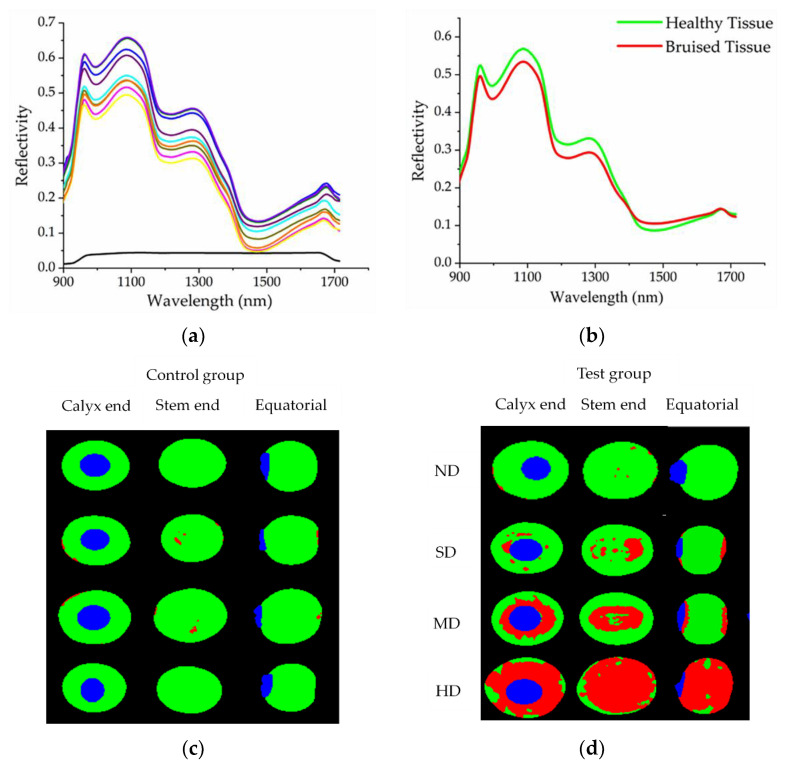
Spectral curve image of blueberries and hyperspectral classification image of each damage stage. The spectral curves of blueberries and background plate (**a**), the black curve is the background plate and the other color curves are the different positions on the blueberry fruit. The spectral curves of blueberry healthy tissue and damaged tissue (**b**). Hyperspectral classification images of the control group (**c**) and test group (**d**).

The spectral information of different regions in the hyperspectral image was extracted. Figure 5a shows the spectral information of the blueberry tissue and the background. The black line at the bottom represented the spectral curve of the background, while the curves of other colors represented the blueberry tissue. Different color curves represented different parts of the blueberries. The spectral curves of blueberry tissue were significantly different from that of the background. The background curve was relatively flat and had low values. In contrast, the blueberry tissue spectral curves were fluctuating more with higher overall values. The reason for this phenomenon was that the reflectivity of blueberries and background plates to light was different. This proved that the spectral image could distinguish blueberries from the background and also showed the reliability of the spectral image. Figure 5b shows the spectral information of healthy and bruised areas of blueberries. The red curve represented the bruised tissue, and the green curve was the healthy tissue. The overall trend of the two curves was consistent, but the overall value of the spectral curve of healthy tissue was larger than that of the bruised spectrum. The reason for this phenomenon may be the presence of water and other nutrients between cells when the blueberry cells rupture, resulting in different reflectivity between healthy and bruised tissues. Especially at 1200 nm, the difference in reflectance between healthy and bruised tissue was the largest. This demonstrated the reliability of 1200 nm for blueberries to differentiate between healthy and bruised tissue.

The image of the bruised blueberries after classification clearly showed the area where each pixel of the blueberries was located. In the classified image, the red area represented the bruised part, the green area represented the healthy part, and the blue area was the calyx. Blueberry bruise damage caused by different compression stages was visually displayed through the processed hyperspectral image. The trend of bruised areas in different stages of the image was consistent with the trend of bruise damage observed with the naked eye, which proved the reliability of the image to detect bruise damage. Blueberries in the control group were not bruised by the naked eye observation. The tiny plaques representing the bruise damage were found after the image analysis, which proved that the imaging treatment is more sensitive to the bruise damage than the visual observation (Figure 5c). Bruise damage to blueberries in the control group could be minor defects during growth or minor scratches from picking and transportation. Blueberries with tiny defects and scratches cannot be removed manually. According to equation (13), the bruise rate of blueberries was calculated, and blueberries were divided into four bruise damage stages (Figure 5d). The four stages were as follows: no obvious bruise damage (ND), slight bruise damage (SD), moderate bruise damage (MD), and harsh bruise damage (HD). When the compression amount was small, the blueberries suffered less damage. At a compression amount of 0.5 mm, the difference between the compressed and uncompressed blueberries in the classification image was not significant. In addition, when the amount of compression was not large, the bruised area was mainly concentrated in the calyx and stem end. The equatorial part was not bruised. As the amount of compression increased, the bruised area began to spread towards the equator. This was consistent with the change in bruise damage in the finite element simulation. The agreement between the image analysis and the simulation results proved the conjecture of the actual tendency of blueberry bruise development. The bruise spread gradually from the calyx and stem end to the equator, and eventually, the all of the blueberries were completely damaged.

There were noticeable differences in the bruise rates of the blueberries subjected to different degrees of compression. The bruise rates and various parameters corresponding to each bruise damage stage are shown in Table 2. The blueberries had minor damage in the ND stage, according to Table 2 and Figure 5d. By observing the location of the damage in the ND stage, it was speculated that the damage may include both the damage caused by the compression experiment and the existing small damage to the blueberries. These two minor damages were combined into the ND stage. The bruise damage rates based on the classified image were about 7% or less. The bruised area of blueberries in the SD stage was more obvious and distributed in spots around the calyx and stem end. The speckled appearance may be related to the tiny size of the blueberries. It may also be caused by the inconsistency of the internal tissues of blueberries to resist damage. The blueberries under the SD stage were less deformed and suffered less force, but at the same time, the damage rates were lower than 35%. The damage to the blueberries in both the ND and SD stages was very small and did not affect the normal sales of blueberries. In the MD stage, due to the increased stress and deformation of the blueberries, the damaged area had coagulated into a larger block. The edge of the bruised area was irregularly protruding and tended to spread to the equatorial part. In the MD stage, blueberries had a relatively high rate of bruise damage. Maximum bruise damage could reach about 70%. Although the blueberries in the HD stage did not rupture, the large deformation of the blueberries caused the internal tissue to be severely squeezed. Blueberries were seriously damaged at this time and the bruised area occupied most of the area of the blueberries. Meanwhile, blueberries became soft and juicy in a relatively short period and were inclined to crack. Blueberries in the MD and HD stages had larger damaged areas, and the fruits were prone to rupture and had a poor taste. As a result, the probability that the blueberries were prone to lesions during the transportation was greatly increased, which had a greater impact on health. At this time, the blueberries were not suitable for normal sales.

### 3.3. Analysis of Influencing Factors of Bruise Degree

Response surface methodology was utilized to analyze the influencing factors of blueberry bruise damage in the Design-Expert software. The coding of impact factors is listed in Table 3, and the experimental scheme and results are shown in Table 4. ANOVA is used to analyze the effect of three factors on the damage rate, as shown in Table 5. It was found that $X_{1}$, $X_{2}$, $X_{3}$, $X_{1}^{2}$, $X_{2}^{2}$, and $X_{3}^{2}$ had significant effects on the damage rate (*p* < 0.05). The lack of fit was not significant, indicating that the model had a good fit (*p* = 0.7529). The predicted $R^{2}$ was 0.9371, indicating a high regression fit. The regression model for the bruise rate was as follows:

(14) $$Y=9.87+7.69X_{1}-2.70X_{2}+1.88X_{3}-0.48X_{1}X_{2}+0.14X_{1}X_{3}-0.25X_{2}X_{3}+2.04X_{1}^{2}\;-1.61X_{2}^{2}+8.85X_{3}^{2}\;$$

where *Y* is the bruise rate of the blueberries, $X_{1}$ represents the maturity, $X_{2}$ represents the loading speed, $X_{3}$ represents the loading location, $X_{1}X_{2}$ represents the interaction item of maturity and loading speed, $X_{1}X_{3}$ represents the interaction item of maturity and loading location, $X_{2}X_{3}$ represents the interaction item of loading speed and loading location, $X_{1}^{2}$ represents the quadratic item of maturity, $X_{2}^{2}$ represents the quadratic item of loading speed, $X_{3}^{2}$ represents the quadratic term of the loading location.

**Figure 6:**
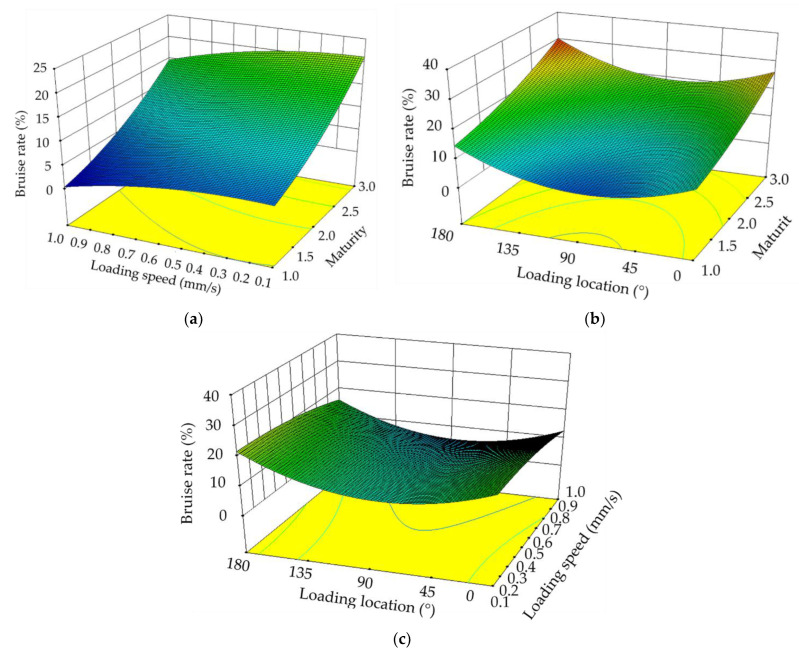
Response surface of influencing factors. Response surface for the effect of loading speed and maturity on bruising rate (**a**), response surface for the effect of loading position and maturity on bruising rate (**b**), and response surface for the effect of loading position and loading speed on bruising rate (**c**).

The response surface results of the regression equation are given in Figure 6. As shown in Table 5, maturity has the greatest impact on the bruise damage rate, followed by loading speed and loading location. The interaction of each factor was not significant. Response surface methods fit the equations to actual data. The equations can be represented graphically to predict the effect of different conditions on the response value. As maturity increased, the blueberry bruise damage rate gradually increased (Figure 6a). The blueberry bruise damage rate was at its greatest when the loading location was at the stem, followed by at the calyx end (Figure 6b). The overall bruise damage rate of the blueberries was the smallest when the loading location was the equator. The reason for this situation may be caused by the geometric shape of the blueberries with a large horizontal size and a small vertical size. The blueberry bruise damage rate initially decreased slowly and then accelerated with increasing loading speed (Figure 6c). The reason for this phenomenon may be that the slower the loading speed, the less elastic recovery of the blueberries. When the loading speed was faster, the elastic recovery ability of the blueberries was also faster.

The model was optimized and analyzed accordingly. The optimization parameters were determined for immature blueberry fruit, a compression speed of 0.96 mm/s, and a compression location of 86°. The corresponding blueberry contusion rate was 1.04%. To verify the accuracy of the response surface optimization, the optimal parameters were selected for experiments. In this experiment, the influence of environmental factors was ignored, and 20 groups of experiments were repeated to eliminate random errors. Blueberry bruise damage was detected by the near-infrared hyperspectral system, and the bruise damage rate was calculated by ENVI. The test proved that the bruise damage rate of blueberries was 1.1%, which was basically in line with the predicted value.

## 4. Conclusions

The main purpose of this study was to classify blueberries with different bruise damage levels and to explore the effects of different influencing factors on blueberry bruise damage. Based on the measurement results of the blueberry geometric dimensions, SOLIDWORKS was used to construct a three-dimensional model of the blueberries. The parameters obtained from the blueberry compression experiment were used in ABAQUS to perform a finite element explicit dynamic simulation. The damage location of the blueberries was predicted, and the bruise damage degree was preliminarily classified as mild and severe. The simulation results showed that the maximum force location of the blueberries was at the calyx end and stem end, and the minimum force location was at the equator. In addition, the simulation results also show the internal stress change trend of the blueberries during the compression process. This started from the calyx and stem end, spread to the entire center column, and then spread to the equator.

The near-infrared hyperspectral reflectance imaging system was used to solve the problem that blueberry bruise damage was difficult to observe and reveal the degree of bruising damage. SVM was used to train and classify the obtained spectral images, visually showing the blueberry’s bruised area. Through the statistical analysis of each pixel in the classified image, the bruise damage rates of the blueberries under different compression levels were obtained. The blueberries were classified based on their degree of bruising damage. The results showed that the bruising rate in the ND stage was 0–7%, the bruising rate in the SD stage was 7–35%, the bruising rate in the MD stage was 35–70%, and the bruising rate in the HD stage was 70–100%. According to response surface analysis, the most influential factor on the bruising rate was maturity, followed by loading speed and loading location. The optimized parameter maturity was immature, the compression speed was 0.96 mm/s, and the compression location was 86°. The actual damage rate was 1.1%. This study provides a theoretical basis for achieving accurate blueberry grading. On a production line of high speed, blueberries can be better classified through image recognition technology. Then, cooperating with the sorting equipment to achieve the rapid and fine sorting of blueberries, better products can be provided to meet customer requirements.

## Figures and Tables

**Table 1:** Value of *K* for various of θ.

θ	50	55	60	65	70	75	80	85	90
cosθ	0.6428	0.5736	0.5000	0.4226	0.3420	0.2588	0.1736	0.0872	0.0000
*K*	1.198	1.235	1.267	1.293	1.314	1.331	1.342	1.349	1.351

**Table 2:** Bruise grades for blueberries.

Degree of Bruise	Bruise Rate (%)	Compression刘Distance (mm)	Load (N)
ND	0–7	0.0–0.5	0.00–1.17
SD	7–35	0.5–2.5	1.17–7.26
MD	35–70	2.5–4.0	7.26–12.27
HD	70–100	4.0–5.0	12.27–16.22

ND: no obvious bruise damage; SD: slight bruise damage; MD: moderate bruise damage; HD: harsh bruise damage.

**Table 3:** Codes of the factors.

Codes	Maturity	Speed (mm/s)	Load Location (°)
−1	1	0.1	0
0	2	0.5	90
1	3	1.0	180

**Table 4:** Experiment schemes and results.

No.	$X_{1}$	$X_{2}$	$X_{3}$	*Y*
1	−1	−1	0	5.06
2	1	−1	0	20.31
3	−1	1	0	1.13
4	1	1	0	14.69
5	−1	0	−1	10.93
6	1	0	−1	27.12
7	−1	0	1	14.69
8	1	0	1	31.42
9	0	−1	−1	18.13
10	0	1	−1	12.57
11	0	−1	1	22.13
12	0	1	1	15.63
13	0	0	0	10.35
14	0	0	0	11.76
15	0	0	0	9.18
16	0	0	0	11.32
17	0	0	0	8.13

**Table 5:** ANOVA of the bruise rate.

Items	Sum of Squares	Degree of Freedom	Mean Square	*F*-Value	*p*-Value
Model	928.07	9	103.12	61.07	<0.0001 ***
$X_{1}$	470.18	1	470.18	278.43	<0.0001 ***
$X_{2}$	58.37	1	58.37	34.57	0.0006 ***
$X_{3}$	27.99	1	27.99	16.58	0.0047 **
$X_{1}X_{2}$	0.91	1	0.91	0.54	0.4857
$X_{1}X_{3}$	0.073	1	0.073	0.043	0.8413
$X_{2}X_{3}$	0.25	1	0.25	0.15	0.7139
$X_{1}^{2}$	17.48	1	17.48	10.35	0.0147 *
$X_{2}^{2}$	10.54	1	10.54	6.24	0.0411 *
$X_{3}^{2}$	330.13	1	330.13	195.50	<0.0001 ***
Residual	11.82	7	1.69		
Lack of fit	2.80	3	0.93	0.41	0.7529
Pure error	9.02	4	2.26		
Cor total	939.89	16			

$R^{2}$ = 0.9874; Adj. $R^{2}$ = 0.9713; Pred. $R^{2}$ = 0.9371; Adeq. Precision = 30.540; *CV* = 9.03%. The Pred. $R^{2}$ of 0.9371 is in reasonable agreement with the Adj. $R^{2}$ of 0.9713. Adeq Precision measures the signal to noise ratio. A ratio greater than 4 is desirable. The ratio of 30.540 indicates an adequate signal. This model can be used to navigate the design space. *: *p* < 0.05, the difference is significant. **: *p* < 0.01, the difference is very significant. ***: *p* < 0.001, the difference is extremely significant.

## Data Availability

Data is contained within the article.
